# Experimental Study of Low-Cycle Fatigue and Recovery of Polymer Blends for Viscous Damping Walls

**DOI:** 10.3390/polym18091022

**Published:** 2026-04-23

**Authors:** Songhang He, Feifei Sun, Defeng Xu, Xiangjun Wu

**Affiliations:** 1 College of Civil Engineering, Tongji University, Shanghai 200092, China; 2310064@tongji.edu.cn; 2 State Key Laboratory of Disaster Reduction in Civil Engineering, Tongji University, Shanghai 200092, China; 3 College of Engineering and Technology, Southwest University, Chongqing 400715, China; wxj19960525@swu.edu.cn

**Keywords:** viscous material, low-cycle fatigue, recovery property, viscous damping wall

## Abstract

The viscoelastic materials used in traditional viscous damping walls (VDWs) typically exhibit high storage moduli, which tend to exacerbate the structural response of adjacent components during earthquakes. Furthermore, existing studies are mostly limited to small-strain characterization and lack investigation into the macroscopic mechanical recovery characteristics of materials under mainshock-aftershock sequences. To overcome these limitations, this study introduces silicone oil (SO) as a softener to prepare a novel viscoelastic polymer blend (PIB-B12-SO). Utilizing a customized self-stabilization dynamic sandwich-type shear (S-DSTS) device, the macroscopic dynamic mechanical behavior of the blend was systematically evaluated, focusing on its low-cycle fatigue and rest-recovery characteristics. The results indicate that the addition of SO effectively reduces the storage modulus and significantly enhances the loss factor of the blend. Notably, at a mixing ratio of 1:4 (SO: PIB-B12), the loss factor increased by 65.6% compared to pure PIB-B12. Furthermore, the introduction of SO effectively suppresses the degradation of the loss modulus under cyclic loading and promotes viscous recovery during the rest periods. The silicone oil blend modification successfully optimizes the macroscopic viscoelastic properties of PIB-B12, significantly enhancing the energy dissipation stability of the material under low-cycle fatigue and interval loading.

## 1. Introduction

Earthquakes pose a persistent threat to the safety and structural integrity of building structures, particularly when they are subjected to strong ground motions and mainshock–aftershock sequences. To mitigate seismic damage, various energy dissipation devices have been developed and widely implemented in structural engineering. Among them, viscous damping walls (VDWs) have attracted increasing attention due to their favorable seismic performance and compatibility with architectural layouts [1,2,3]. VDWs can effectively reduce the seismic responses of buildings, thereby enhancing overall seismic resilience, with applications ranging from modern frame structures to the reinforcement of traditional masonry structures [4].

In recent years, to meet the increasingly stringent demands of structural vibration control under complex and multidirectional seismic sequences, the design and application of VDWs have rapidly evolved [5]. Recent studies have focused on the spatial optimization of VDWs and the development of novel configurations to maximize energy dissipation efficiency within limited structural spaces [6]. The seismic performance of VDWs is largely governed by the rheological and mechanical properties of the viscoelastic materials used as the core energy dissipation medium [7]. These materials are typically semi-solid polymers, whose mechanical properties exhibit pronounced frequency- and strain-dependent characteristics. Consequently, the effectiveness of VDWs relies not only on their structural configuration but also, more critically, on the dynamic mechanical properties of these damping materials.

Under seismic loading conditions, viscoelastic materials are subjected to complex cyclic and time-dependent demands [8,9,10]. The energy dissipation mechanism of viscoelastic materials is determined by the interplay between their elastic response (storage modulus, $G^{\prime }$) and viscous response (loss modulus, $G^{\prime \prime }$). A higher loss factor (μ) is generally desired to enhance energy dissipation, a critical evaluation of currently employed VDW materials reveals a tendency toward high storage moduli. This characteristic increases the global lateral stiffness of the structure. Consequently, during seismic events, this added stiffness can induce larger structural responses and transfer higher internal forces to adjacent connected members (e.g., beams and columns) [11,12,13]. Therefore, a primary challenge is to moderate the storage modulus while maintaining dissipation capacity to prevent adverse impacts on adjacent components.

Such materials have been extensively studied in other engineering fields but remain relatively underexplored in civil engineering [14,15,16,17]. Furthermore, existing research on VDW polymers exhibits specific limitations regarding testing conditions. First, many studies characterize mechanical properties primarily under small shear strains. However, under severe earthquake excitations, the shear strain imposed on VDW materials frequently exceeds 1.0 [3,18]. Thus, small-strain data may not fully represent the material’s actual nonlinear behavior. Second, while low-cycle fatigue has been investigated, the performance recovery of these materials after varying quiescent times remains underexplored. For realistic mainshock–aftershock scenarios, understanding this time-dependent recovery is essential for accurately evaluating the continued resilience of the structure [14,15,16,19,20,21,22].

To tailor these macroscopic properties, incorporating physical plasticizers into polymers is an effective strategy [23,24]. For highly viscous matrices like pure polyisobutylene-B12 (PIB-B12), introducing silicone oil (SO) acts as an effective softening agent to reduce the overall storage modulus. This macroscopic softening yields a more compliant medium, enabling it to accommodate the repeated large-amplitude shear deformations required for seismic energy dissipation. Furthermore, while excessive softening might conventionally reduce the total dissipation capacity, optimizing the SO mass fraction provides a pathway to balance stiffness and dissipation. As observed in other structural damping materials [22], blending SO can moderate the storage modulus while maintaining or enhancing the loss factor, thereby optimizing the material for realistic seismic applications.

Addressing the aforementioned research gaps, the novelty of this study is established through three key aspects. First, a viscoelastic polymer blend (PIB-B12-SO) is developed by incorporating SO into a PIB-B12 matrix. This deliberately lowers the elastic energy storage capability (storage modulus) to reduce adverse load transfer to adjacent structural components, while simultaneously enhancing viscous energy dissipation. Second, dynamic shear tests are conducted at large strain amplitudes (up to 2.0) to better replicate actual seismic demands. Third, loading protocols with varying rest intervals are designed to investigate the mechanical recovery capabilities under simulated mainshock–aftershock conditions.

To systematically evaluate these properties across various loading frequencies, shear strain amplitudes, and blend compositions, a custom Self-Stabilization Dynamic Sandwich-Type Shear (S-DSTS) device is utilized. This device crucially overcomes existing limitations in testing the long-term static recovery of semi-solid materials. Based on this comprehensive approach, the primary objective of this study is to develop and experimentally substantiate the optimal composition of a polymer composite material based on polyisobutylene with the addition of silicone oil, which ensures maximum effective energy absorption and stability of characteristics under cyclic loads in structures of seismic protection of buildings. Furthermore, based on the experimental data, an evolution model characterizing the variation in macroscopic material parameters under cyclic loading is proposed. Ultimately, this work provides both a theoretical basis for parametric optimization and reliable fundamental material parameters for the advanced application of PIB-B12-SO blends in VDWs.

## 2. Experimental

### 2.1. Materials

Polyisobutylene (PIB-B12, commercial grade Oppanol B12 SFN) and silicone oil(SO) were purchased from BASF SE (Ludwigshafen, Germany) and Wacker Chemie AG (Munich, Germany), respectively. All materials were used as received without further purification. Macroscopically, the pristine PIB-B12 presents as a clear to slightly turbid, colorless to pale-yellow highly viscous semi-solid. The primary physicochemical properties of the PIB-B12 matrix, as provided by the manufacturer, are summarized in Table 1. The inherent properties of the pristine PIB-B12 dictate the formulation strategy of this study. Its relatively high viscosity-average molecular weight induces severe polymer chain entanglement, fundamentally resulting in an excessively high storage modulus. Therefore, the incorporation of a liquid plasticizer, such as SO, is physically imperative.

In this study, the preparation of the viscoelastic polymer blends (PIB-B12-SO) followed the well-established protocols for classical immiscible polymer model systems [25,26]. First, the PIB-B12 matrix was preheated to 80 °C to enhance its fluidity. Subsequently, the preheated PIB-B12 and SO at specific mass ratios were subjected to continuous mechanical mixing under constant heating until the blend completely transformed into a white creamy mixture. Finally, referencing similar literature on polyisobutylene blends [27], the vacuum degassing temperature was set to 80 °C. This method of blend preparation has been used previously by [28,29] and proved to be adequate.

### 2.2. Fundamental Mechanical Parameters

**Figure 1:**
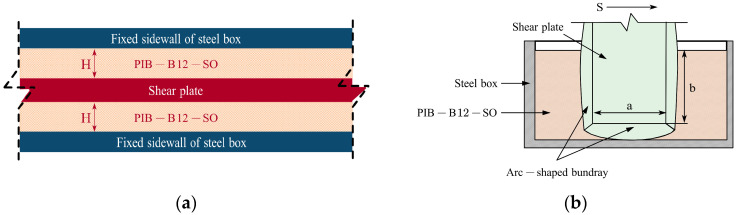
Schematic diagram of dynamic sandwich-type shear test: (**a**) top view and (**b**) front view.

The S-DSTS test device is illustrated in Figure 1. A shear plate is embedded within the PIB-B12-SO blend, which is firmly adhered to the plate surfaces to ensure a no-slip boundary condition.

The shear plate is subjected to a sinusoidal excitation S(t) = u·sin(ωt), while the bottom steel plate remains fixed. As the actuator displaces the shear plate, it forces the PIB-B12-SO material to deform, thereby generating the shear strain γ. The resulting viscoelastic resistance acts on both sides of the shear plate, producing a total macroscopic response force *F*. The clearance between the shear plate and the sidewall plates is denoted as *H*. The damping force *F* and displacement *S* are continuously recorded by the built-in sensors of the actuator. The shear strain and shear stress are calculated as follows (Equations (1) and (2)):

(1) $$\tau =\frac{F}{A}$$

(2) $$\gamma =\frac{S}{H}$$

where A = φ· (2ab), a denotes the calculated shear area, *b* is the computed depth, and φ is an amplification factor that accounts for the area of the arc-shaped boundary. In this study, φ is set to 1.18 [22].

### 2.3. Viscoelastic Property Parameters

According to Boltzmann’s superposition theory [30], the shear stress can be expressed as (Equation (3)):

(3) $$\tau =G^{\prime }(\omega )\gamma (t)+\mu^{\prime }(\omega )\dot{\gamma }(t)$$

where τ is the shear stress $(N/m^{2})$, γ(t) is the shear strain, $\dot{\gamma }\left(t\right)\;=\;\frac{u}{H}\omega \cdot \cos(\omega t)$ is the strain rate, $G^{\prime }(\omega )$ is the storage modulus $(N/m^{2})$, and $\mu^{\prime }(\omega )$ is the dynamic viscosity $(N\cdot s/m^{2})$. The loss modulus $G^{\prime \prime }(\omega )\;(N/m^{2})$ and storage viscosity $\mu^{\prime \prime }(\omega )\;(N\cdot s/m^{2})$ can be calculated by (Equations (4) and (5)):

(4) $$\mu^{\prime \prime }(\omega )=\frac{G^{\prime }(\omega )}{\omega }$$

(5) $$G^{\prime \prime }(\omega )=\omega \mu^{\prime }(\omega )$$

The storage modulus $G^{\prime }\;$ is determined from the linear portion of the stress-strain hysteresis loop using the radial linear regression method [21], an approach well-validated for processing the experimental data of polymeric materials. Based on the measured τ and $G^{\prime }$ the viscous shear stress ${\;\tau }_{V\;}$ is calculated as (Equation (6)):

(6) $$\tau_{V}=\tau -G^{\prime }\gamma$$

Given that highly viscous polymer blends typically exhibit non-Newtonian rheological behavior under large deformations, a nonlinear relationship exists between the viscous shear stress and the strain rate. This relationship is often expressed as (Equation (7)):

(7) $$\tau_{V}=\mu^{\prime }\cdot {\dot{\gamma }}^{\alpha }$$

where α  is the velocity exponent. The total shear stress can be expressed as the sum of elastic and viscous components (Equation (8)):

(8) $$\tau =\tau_{V}+\tau_{E}=\mu^{\prime }\cdot {\dot{\gamma }}^{\alpha }+G^{\prime }\gamma$$

where ${\;\tau }_{E\;}$ is the elastic shear stress.

The loss factor η is determined from the energy balance within a single loading cycle (Equation (9)):

(9) $$\eta =\frac{E_{D}}{2\pi E_{\mathrm{Tot}}}$$

where $E_{D}$ is the dissipated energy obtained from the hysteresis area, and $E_{\mathrm{Tot}}\;=\;0.5G^{\prime }\gamma_{\max}^{2}$ is the total mechanical energy. In this study, $\gamma_{\max}$ is the maximum strain during the loading process.

The loss modulus and velocity exponent can be evaluated using the pivotal point method (Equations (10) and (11)):

(10) $$\ln(\tau_{V_{\gamma_{\max}}}\omega )=\ln(G^{\prime \prime })+\alpha \ln({\dot{\gamma }}_{\max})$$

(11) $$\ln(\tau_{V_{\gamma_{0.5\max}}}\omega )=\ln(G^{\prime \prime })+\alpha \ln({\dot{\gamma }}_{0.5\max})$$

where ${\dot{\gamma }}_{\max}$ is the maximum strain rate, ${\dot{\gamma }}_{0.5\max}\;=\;0.5{\dot{\gamma }}_{\max}$, $\tau_{V_{{\dot{\gamma }}_{\max}}}$ is the stress at $\dot{\gamma \;}={\dot{\;\gamma }}_{\max}$, and $\tau_{V_{{\dot{\gamma }}_{0.5\max}}}$ is the stress at $\dot{\gamma }\;=\;{\dot{\gamma }}_{0.5\max}$.

### 2.4. Performance Evolution Parameters

#### 2.4.1. Low-Cycle Fatigue Variation Ratio

To quantify the low-cycle fatigue behavior of PIB-B12-SO blends, the low-cycle fatigue variation ratio is defined based on the absolute relative change in the material parameters with increasing loading cycles. The variation ratio is expressed as (Equation (12)):

(12) $$\xi^{n}=\frac{\left|Q_{30}^{n}-Q_{1}^{n}\right|}{Q_{1}^{n}}$$

where $Q_{i}^{n}$ denotes the value of the storage modulus, loss modulus, or loss factor at the *i*-th loading cycle under test case *n*. $Q_{5}^{1}$ refers to the parameter value recorded at the 5th cycle of Case 1. For the storage modulus $G^{\prime }$ and loss modulus $G^{\prime \prime }$, a larger variation ratio ξ  indicates a more pronounced degradation under cyclic loading. For the loss factor, an increasing ξ reflects an enhancement in energy dissipation capacity.

#### 2.4.2. Recovery Coefficient

To characterize the restoration of the macroscopic rheological properties following a quiescent rest interval, the recovery coefficient is expressed as (Equation (13)):

(13) $$\psi^{n}=\frac{Q_{1}^{n}-Q_{1}^{1}}{Q_{30}^{1}-Q_{1}^{1}}$$

For the $G^{\prime }$ and $G^{\prime \prime }$ the recovery coefficient quantifies the recovery of elastic response and viscous responses after the resting period:

0 < Ψ < 1 indicates partial recovery,Ψ = 0 denotes full recovery, Ψ = 1 stabilized fatigued state with no immediate recovery, and Ψ > 1 implies continued degradation.

For the loss factor, a larger value Ψ indicates a more significant improvement in the material’s damping capability.

#### 2.4.3. Silicone Oil Average Softening Ratio

To investigate the influence of SO content on the mechanical properties, an average softening ratio is defined. This parameter is calculated using the storage modulus, loss modulus, and loss factor. The formula is as follows (Equation (14)):

(14) $$\beta^{n,j}=\frac{\sum_{i=1}^{30}\left(\frac{Q_{ij}^{n}-Q_{i1}^{n}}{Q_{i1}^{n}}\right)}{30}$$

where *j* denotes the specific blend composition (1, 2, 3, 4, 5). Specifically, *j* = 1 represents the mixing ratio of 1:0 (PIB-B12:SO).

### 2.5. Test Device

**Figure 2:**
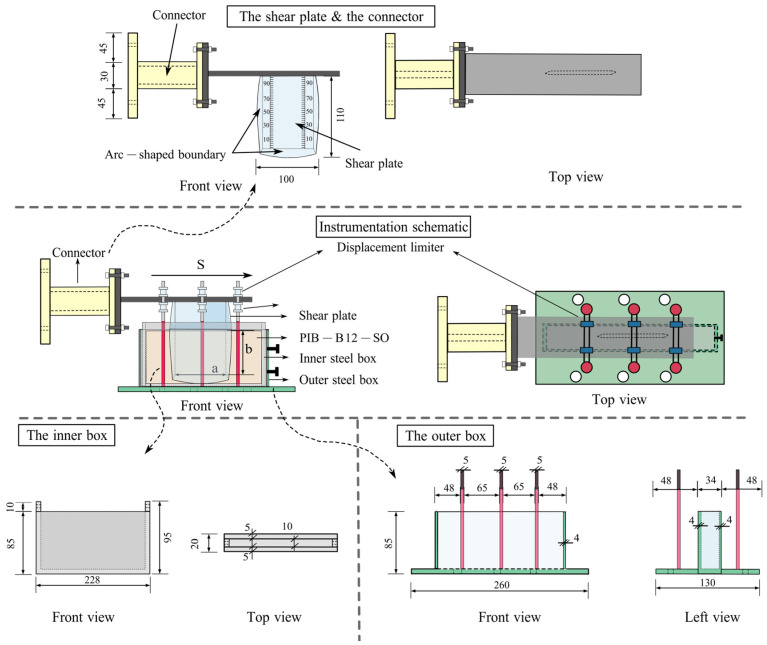
Components and assembly schematic of the S-DSTS device.

A self-stabilizing dynamic sandwich shear (S-DSTS) device was designed to characterize the fatigue and structural recovery properties of semi-solid polymer blends, effectively addressing the limitations of conventional rheological instruments in evaluating the quiescent recovery of highly viscous materials. The core assembly comprises an outer steel box, an inner steel box, a shear plate, and a connector. The individual components are illustrated in Figure 2.

**Figure 3:**
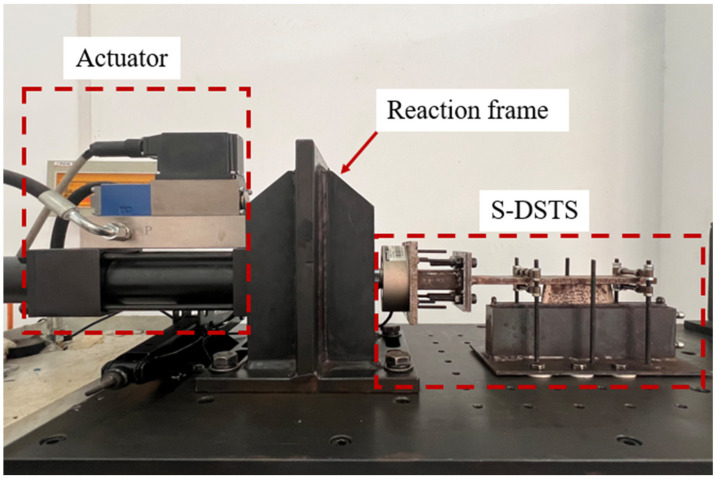
Actual installation of the S-DSTS device.

To ensure measurement accuracy, the outer steel box is securely anchored to the test bed via integrated bolt holes, while the inner steel box is nested and bolted to the outer casing to eliminate parasitic relative displacement. The shear plate features sharp arc-shaped boundaries on three material-contact faces to minimize edge effects and eliminate compressive stress (squeezing effect) during oscillatory shear deformation. The connector links the shear plate to a linear actuator using high-strength bolts. To maintain pure horizontal displacement, steel roller limiters are positioned on both sides of the shear plate to constrain vertical motion while minimizing frictional drag. The polymer sample (e.g., PIB-B12-SO) was uniformly cast into the 4 mm gap between the shear plate and the inner box walls. The configuration of the S-DSTS device, including its instrumentation schematic and physical installation, is depicted in Figure 2 and Figure 3.

The testing assembly was mounted within a rigid reaction frame located at the Structural Laboratory of Tongji University. Since the PIB-B12-SO blend is intended for use as the core energy dissipation medium in viscous damping walls (VDWs), the loading protocols were strictly formulated in accordance with the testing provisions of the American structural seismic design standard, ASCE/SEI 7-16 [31]. Dynamic tests were conducted under displacement-controlled sinusoidal oscillatory shear. The resulting damping force and displacement signals were recorded in real-time by a high-precision data acquisition system integrated within the actuator assembly.

### 2.6. Test Cases

To investigate the effects of silicone oil (SO) content on the energy dissipation capacity and recovery performance of PIB-B12-SO, a series of experimental studies was conducted. The test conditions are shown in Table 2. To ensure statistical reproducibility, each loading condition was independently repeated three times.

The rationale for the specific parameter configurations is as follows. First, the range of SO blending ratios was deliberately selected to uniformly cover the practically viable engineering domain, capturing the material’s physical transition from a relatively stiff semi-solid to a highly compliant blend. Second, the loading frequency range was strategically chosen to encompass the fundamental natural frequencies of most mid- to high-rise building structures. According to structural dynamics theory, the fundamental periods of these buildings typically range from 0.5 s to 5.0 s, corresponding to a frequency band of 0.2 Hz to 2.0 Hz [32]. Simultaneously, standard seismic design response spectra indicate that the primary energy of ground motions is similarly concentrated within this low-to-medium frequency range [33]. Finally, regarding the macroscopic shear strain amplitudes, referencing existing experimental studies on VDWs under severe earthquake excitations reveals that peak shear strains frequently exceed 1.0, occasionally reaching approximately 2.0 under extreme conditions [3]. Therefore, the large-strain protocols adopted herein are explicitly necessary for these severe structural demands.

Furthermore, a multi-stage intermittent rest-recovery test, comprising four sequential loading and quiescent stages, was conducted at a fixed shear strain amplitude of 2.0 and a loading frequency of 1.0 Hz. Based on Table 2, the test conditions can be designated accordingly. For example, VP2-VS1-VF2-RP2 denotes the third rest–recovery loading of a PIB-B12-SO material with a mixing ratio of 5:1, conducted at a shear strain of 0.5 and a loading frequency of 0.6 Hz.

## 3. Results

### 3.1. Low-Cycle Dynamic Fatigue Property of PIB-B12-SO

#### 3.1.1. Effect of Silicone Oil Content

**Figure 4:**
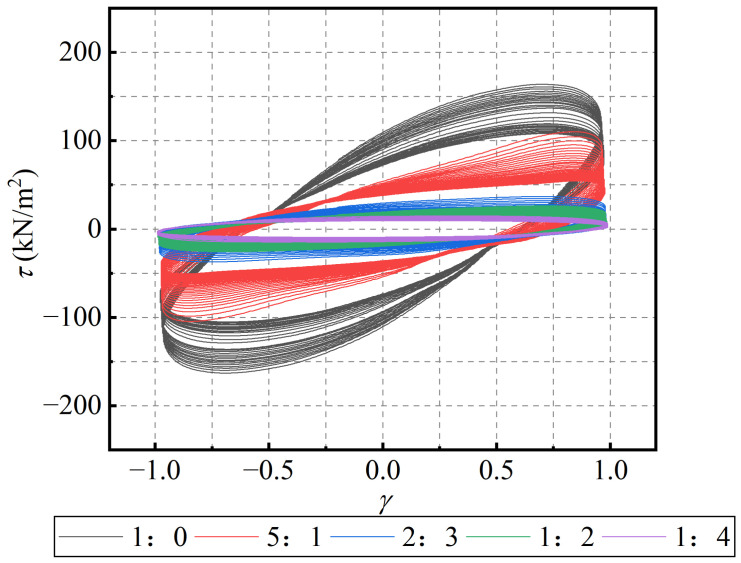
Stress-strain hysteresis loops of the PIB-B12-SO blends under different mass ratios (VS2-VF3).

The effect of material mixing ratio was evaluated through an analysis of the stress-strain hysteresis loops, primary rheological parameters, and the low-cycle fatigue variation ratios. stress-strain hysteresis loops of PIB-B12-SO blends with different mixing ratios, tested at a constant loading frequency of 1.0 Hz and a shear strain amplitude of 1.0, are presented in Figure 4. As shown in the figure, as the SO content increases, the linear stiffness of the hysteresis curves gradually decreases, while the viscous characteristics become more pronounced, leading to a higher ratio of dissipated energy to stored elastic energy. Therefore, the addition of SO to the material significantly reduces the storage modulus while enhancing the energy dissipation capacity, warranting an in-depth investigation.

**Figure 5:**
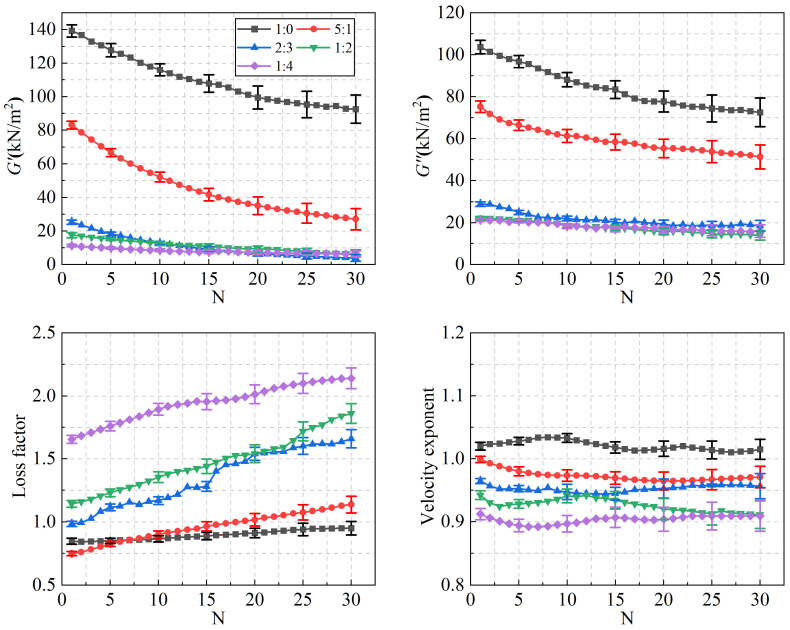
Variation in material parameters with the number of loading cycles for different mixing ratios.

**Figure 6:**
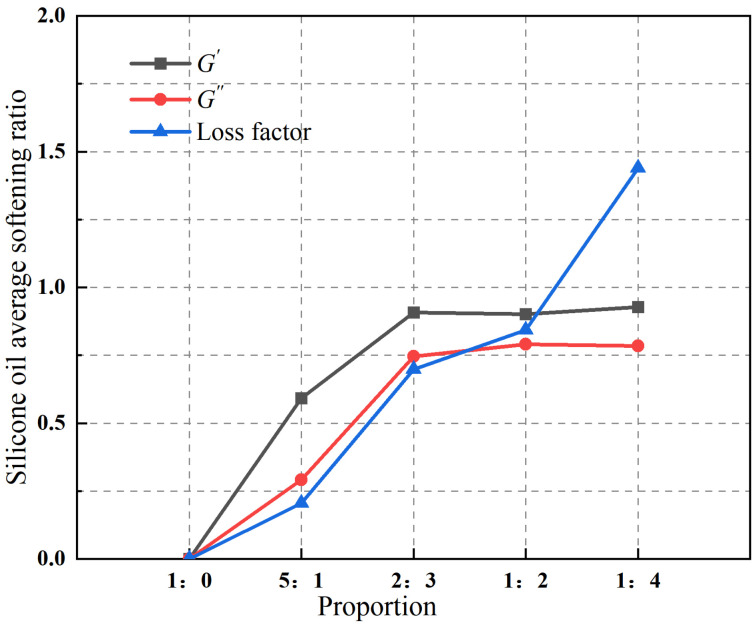
Variation in softening ratios with the number of loading cycles for different mixing ratios.

The evolution of the macroscopic rheological parameters as a function of loading cycles for different blend compositions is illustrated in Figure 5. As the SO content increases, the storage modulus, loss modulus, and velocity exponent of the PIB-B12-SO exhibit an overall decreasing trend. When the mixing ratio reaches 1:2, the rate of decrease for these parameters becomes less pronounced. In contrast, the loss factor increases continuously with increasing SO content. The relationship between the mixing ratio and the average softening ratio of the material parameters is presented in Figure 6. The results indicate that the average softening ratio of the loss factor increases markedly with increasing SO content, whereas the average softening ratios of the storage modulus and loss modulus tend to level off after an initial rapid increase. At a mixing ratio of 1:4, the average softening ratio of the storage modulus is 92.78%, that of the loss modulus is 78.42%, and that of the loss factor reaches 143.92%. This indicates a significant reduction in the storage modulus alongside a pronounced enhancement in the energy dissipation capability.

**Figure 7:**
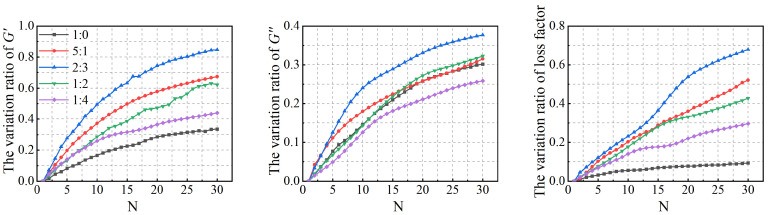
Relationship between low-cycle fatigue variation ratio and material mixing ratio.

The relationships between the low-cycle dynamic fatigue variation ratios and the blend compositions are presented in Figure 7. As shown in the figure, the variation ratios of the storage modulus, loss modulus, and loss factor first increase and then decrease with increasing SO content, reaching a peak at a mixing ratio of 2:3. The 1:4 PIB-B12-SO exhibits more pronounced macroscopic degradation of the storage modulus, which is beneficial for reducing the dynamic stiffness of the VDWs, while maintaining relatively small degradation of the loss modulus, thereby ensuring effective energy dissipation.

For the 1:4 PIB-B12-SO, the variation ratios of the storage modulus and the loss factor are greater than those of the pure PIB-B12 (1:0), while its loss modulus variation ratio is smaller. This behavior is attributed to the fact that the loss modulus dominates over the storage modulus following the addition of SO, reflecting enhanced energy dissipation characteristics.

The slope of the curves relating the parameter variation ratio to the number of loading cycles gradually decreases with increasing cycles, indicating that there is a lower limit of the material degradation, which is consistent with the study of PIB-12.

#### 3.1.2. Effect of Loading Frequency

**Figure 8:**
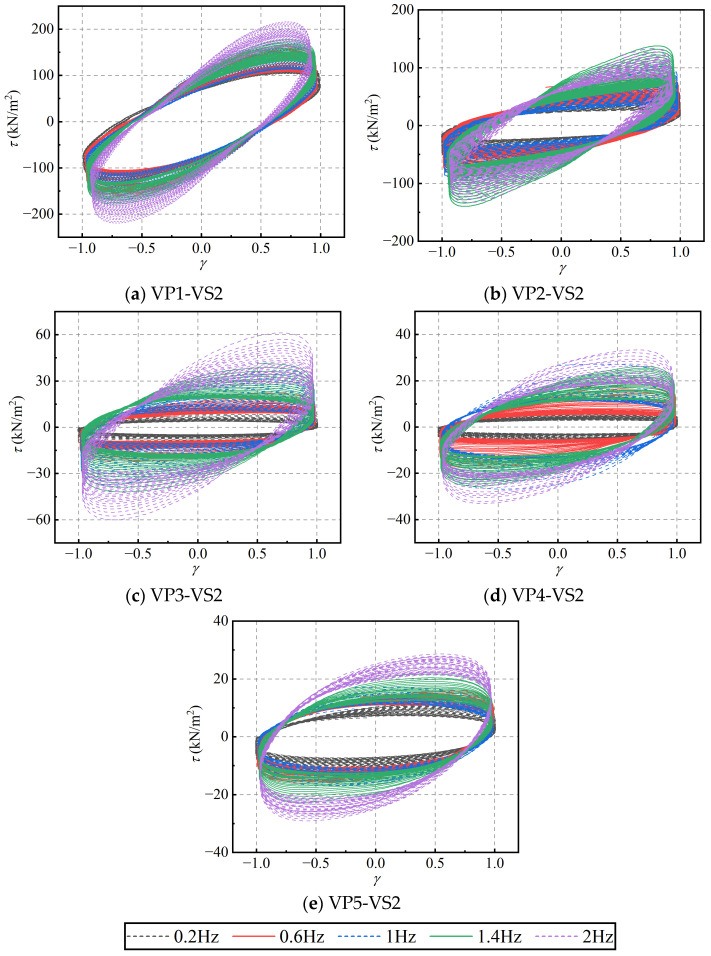
Hysteresis loops of PIB-B12-SO with different mixing ratios under different loading frequencies.

Similarly, the influence of the loading frequency on the macroscopic dynamic response of the PIB-B12-SO blends across different compositions was investigated by analyzing the stress-strain hysteresis loops, rheological parameters, and dynamic fatigue variation ratios. The stress-strain hysteresis loops of the PIB-B12-SO with a strain of 1.0 and different frequencies are shown in Figure 8. It can be observed that as the loading frequency increases, the peak stress, the slope of the hysteresis curve, and the area of the hysteresis loop all increase. These results demonstrate that the PIB-B12-SO blends maintain a robust viscous energy dissipation capacity over a broad spectrum of loading frequencies, with higher frequencies leading to enhanced energy dissipation.

**Figure 9:**
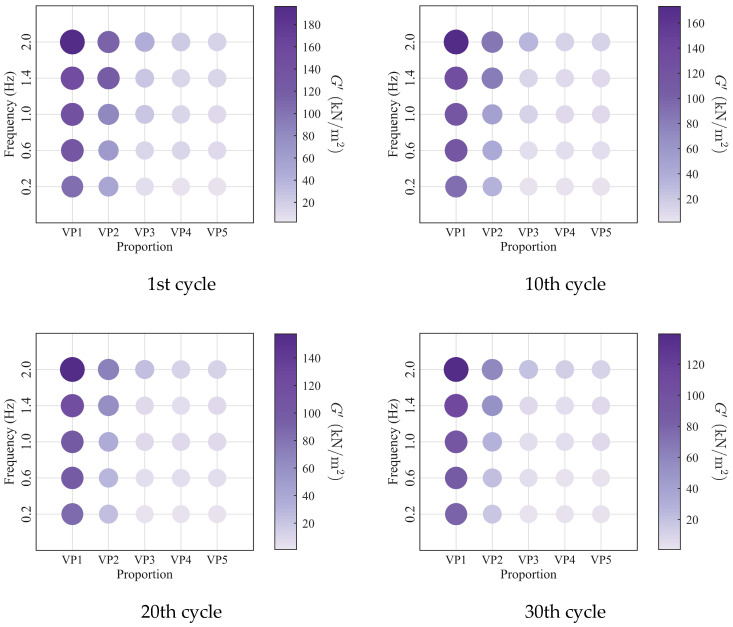
Storage modulus of PIB-B12-SO with different mixing ratios under different loading frequencies.

**Figure 10:**
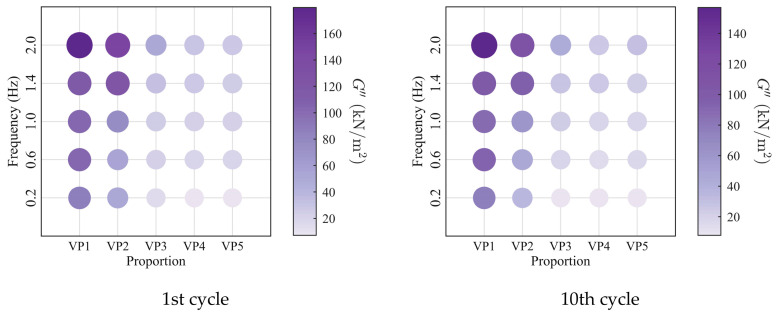

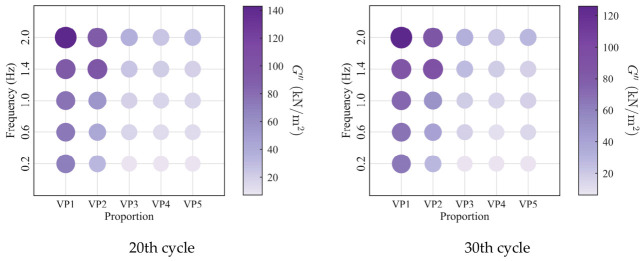
Loss modulus of PIB-B12-SO with different mixing ratios under different loading frequencies.

**Figure 11:**
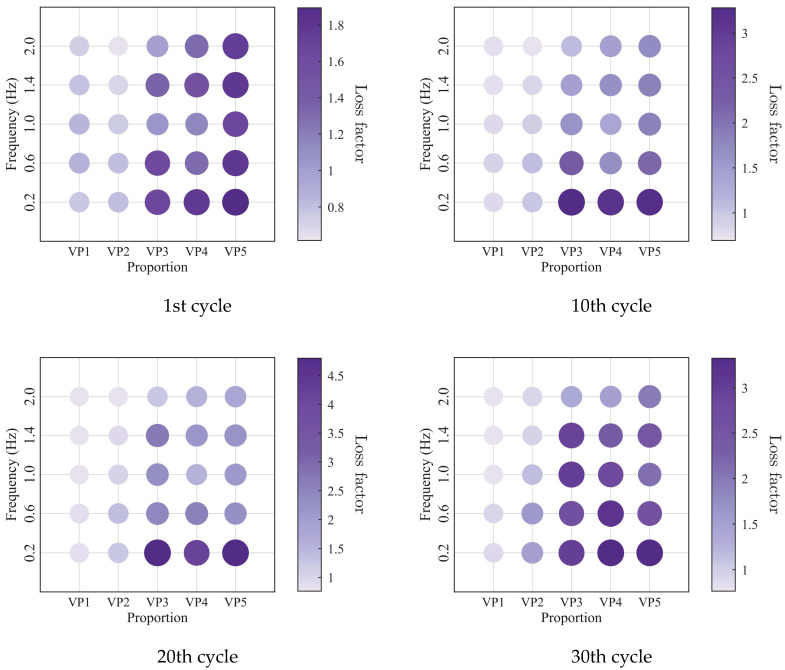
Loss factor of PIB-B12-SO with different mixing ratios under different loading frequencies.

**Figure 12:**
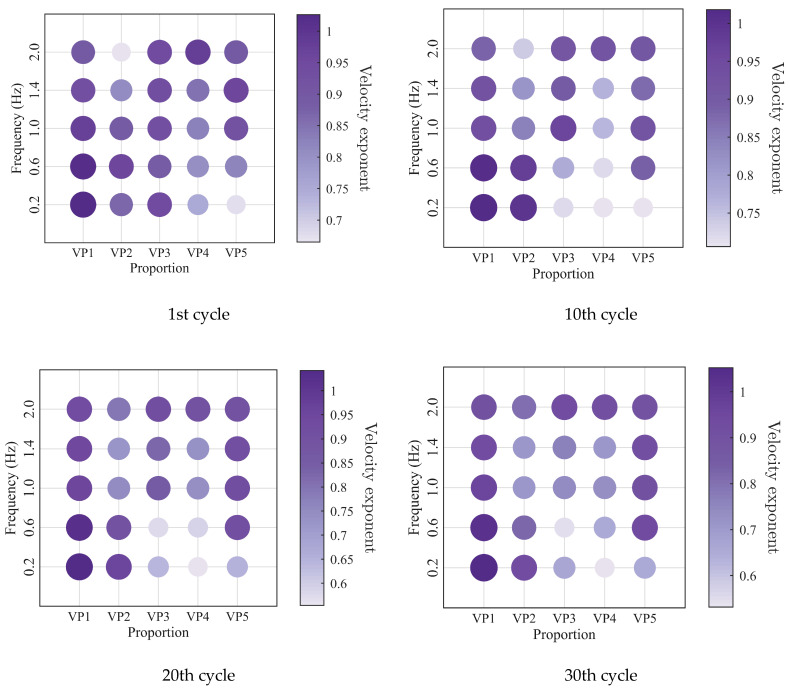
Velocity exponent of PIB-B12-SO with different mixing ratios under different loading frequencies.

To further investigate the evolution of the macroscopic rheological parameters for blends with different mixing ratios under different loading frequencies, the material parameters extracted at the 1st, 10th, 20th, and 30th loading cycles are presented in Figure 9, Figure 10, Figure 11 and Figure 12. At the same loading cycles, both the storage modulus and loss modulus of PIB-B12-SO increase with increasing loading frequency, whereas the loss factor decreases, which is consistent with existing studies [22,34]. Additionally, the loss factor at low frequency (0.2 Hz) consistently exceeds that at higher frequencies, indicating that the energy dissipation capacity of the PIB-B12-SO is more pronounced under low-frequency loading.

**Figure 13:**
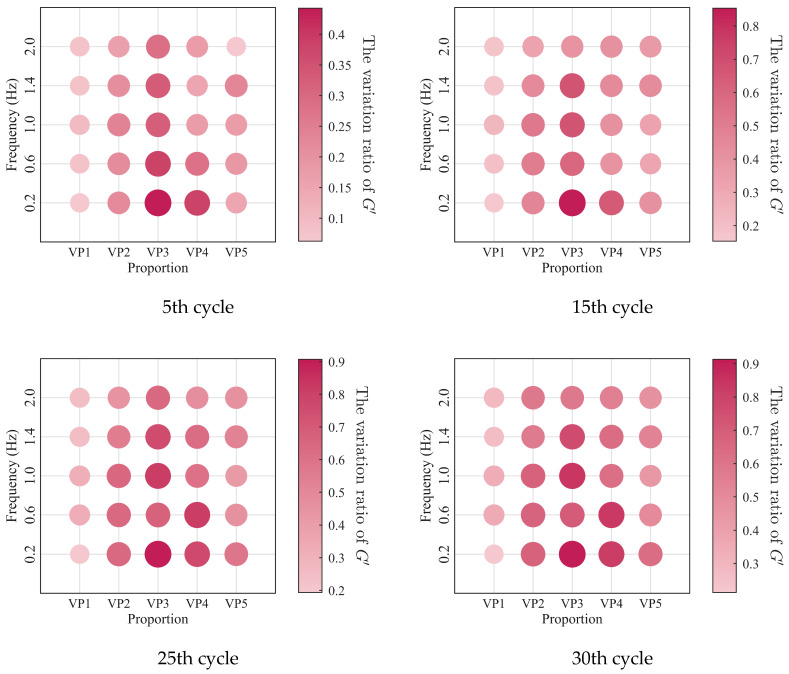
Decay ratio of the storage modulus of PIB-B12-SO with different mixing ratios under different loading frequencies.

**Figure 14:**
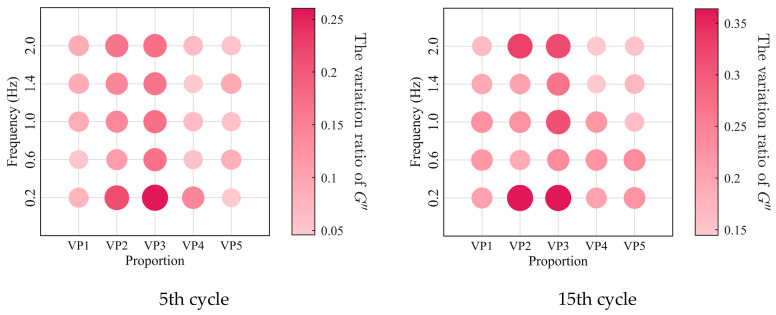

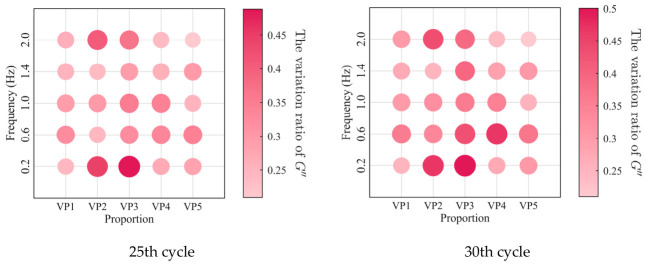
Decay ratio of the loss modulus of PIB-B12-SO with different mixing ratios under different loading frequencies.

**Figure 15:**
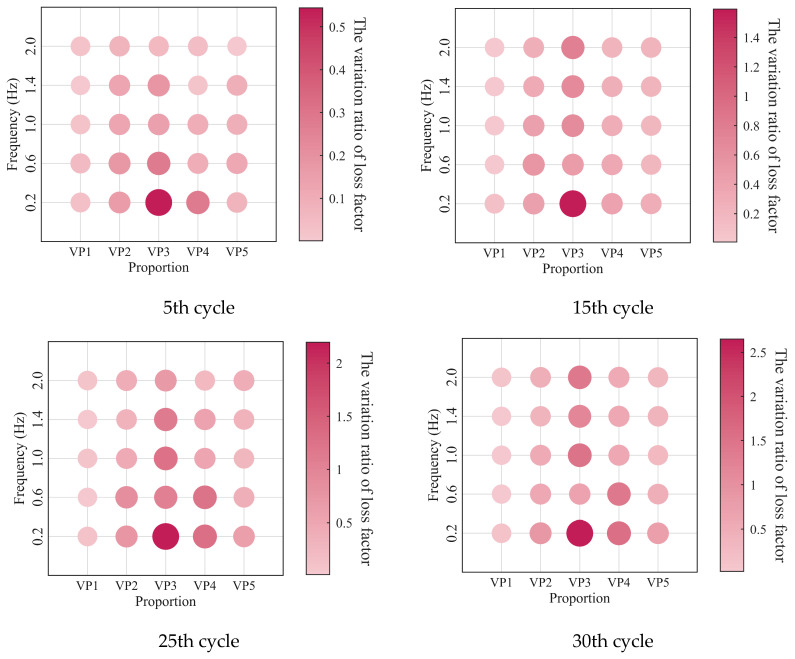
Growth ratio of the loss factor of PIB-B12-SO with different mixing ratios under different loading frequencies.

The low-cycle dynamic fatigue variation ratios under different loading frequencies for various mixing ratios of PIB-B12-SO are shown in Figure 13, Figure 14 and Figure 15. The trend of the low-cycle fatigue variation ratios fundamentally insensitive to variations in the loading frequency, fluctuating only within a narrow margin. This suggests that the loading frequency exerts a limited influence on the dynamic low-cycle fatigue behavior. Regardless of the loading frequency, both the variation ratios increase with the accumulating loading cycles, eventually converging toward an asymptotic plateau. This observation confirms the existence of a boundary limit for the degradation of the storage modulus and loss modulus, as well as for the increase in the loss factor.

#### 3.1.3. Effect of Shear Strain Amplitude

**Figure 16:**
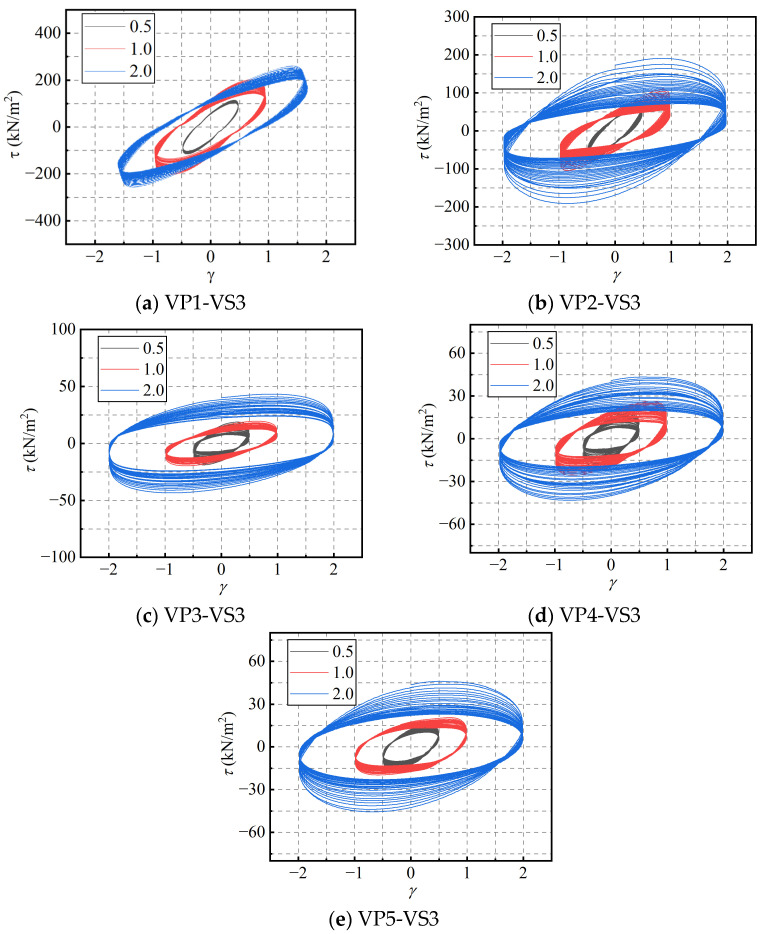
Hysteresis loops of PIB-B12-SO with different mixing ratios under different shear strains.

Furthermore, the stress-strain hysteresis loops, macroscopic rheological parameters, and low-cycle fatigue variation ratios of PIB-B12-SO were analyzed to investigate the effect of different strain amplitudes on its mechanical properties at a loading frequency of 1.0 Hz. The hysteresis loops are shown in Figure 16. It is evident that both the peak stress and the hysteresis area increase significantly with an increasing shear strain amplitude. Accordingly, the energy dissipation capacity of the blends is markedly enhanced under larger shear strain amplitudes. In addition, the dispersion between consecutive hysteresis loops increases at higher strain levels.

**Figure 17:**
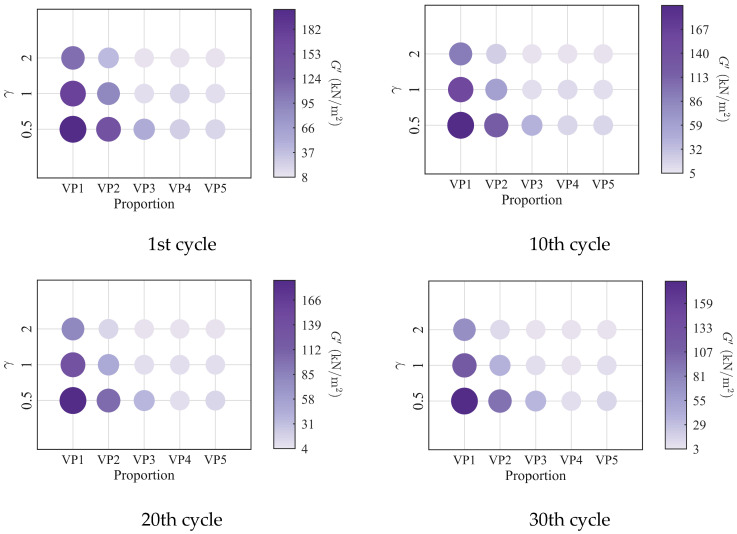
Storage modulus of PIB-B12-SO with different mixing ratios under different shear strains.

**Figure 18:**
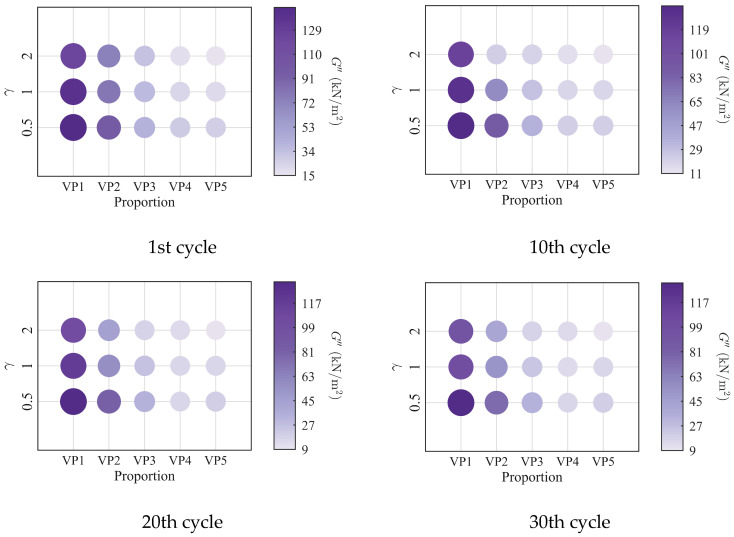
Loss modulus of PIB-B12-SO with different mixing ratios under different shear strains.

**Figure 19:**
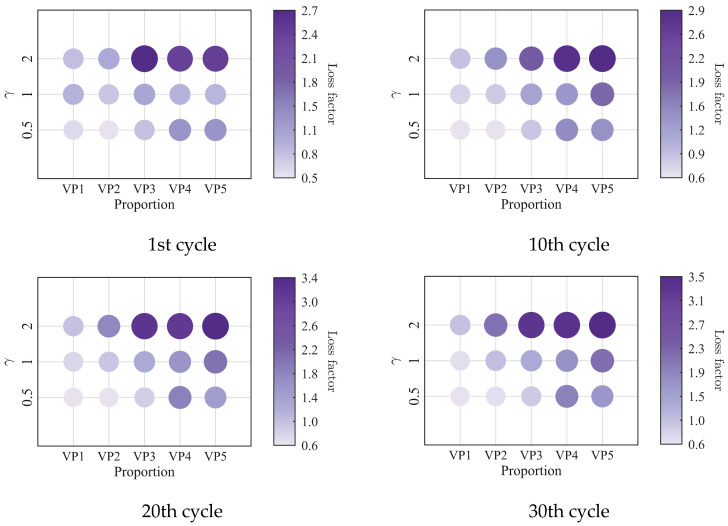
Loss factor of PIB-B12-SO with different mixing ratios under different shear strains.

**Figure 20:**
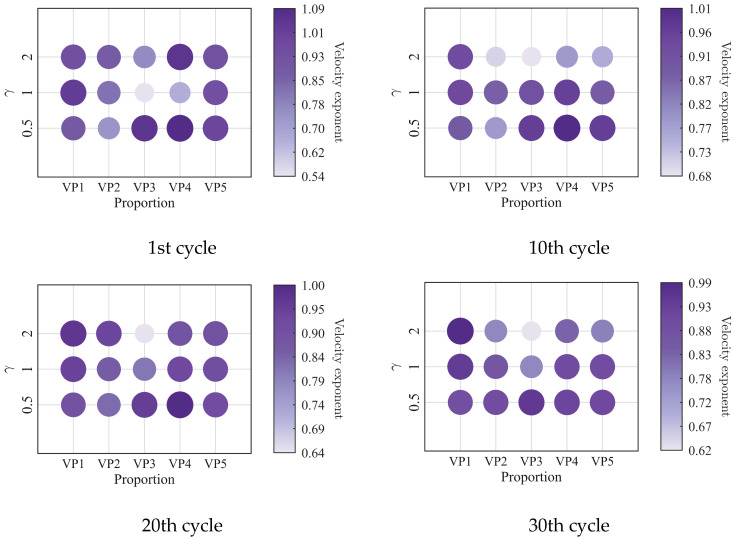
Velocity exponent of PIB-B12-SO with different mixing ratios under different shear strains.

The primary rheological parameters of blends with different mixing ratios under different shear strains are shown in Figure 17, Figure 18, Figure 19 and Figure 20. Both the storage modulus and the loss modulus decrease with increasing strain amplitude, while the loss factor increases, consistent with existing studies [22,34]. It is noteworthy that at a large strain amplitude (i.e., 2.0), the enhancement of the loss factor with the increasing SO content becomes significantly more pronounced.

**Figure 21:**
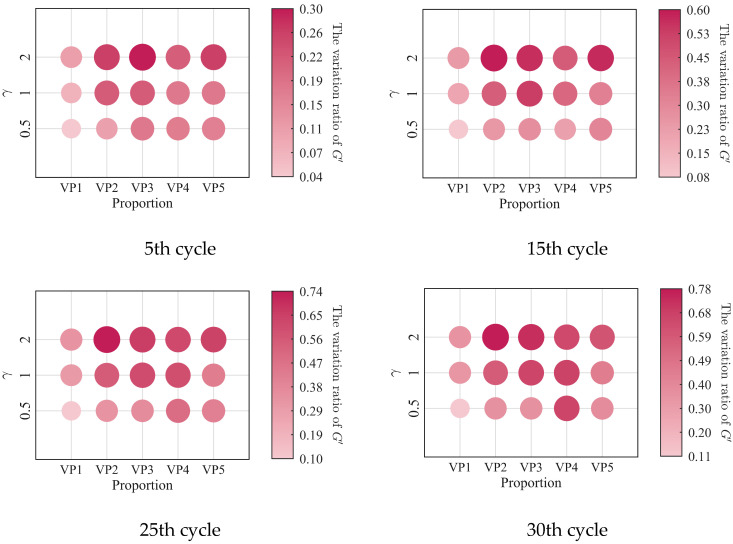
Decay ratio of the storage modulus of PIB-B12-SO with different mixing ratios under different shear strains.

**Figure 22:**
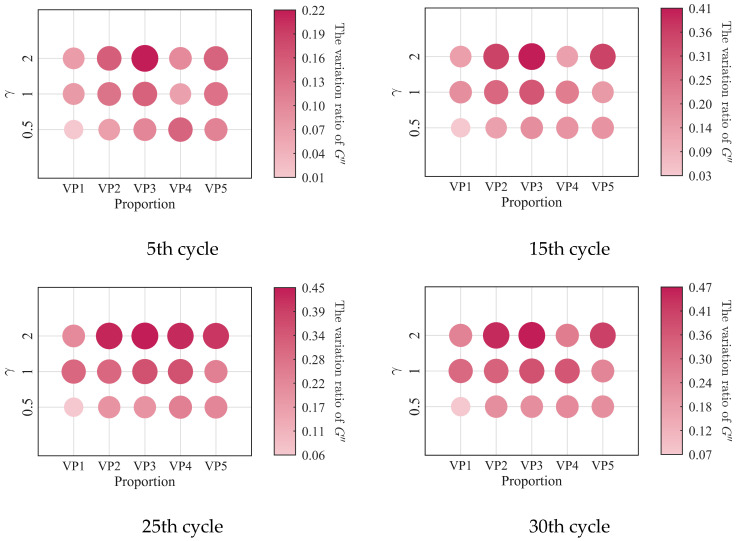
Decay ratio of the loss modulus of PIB-B12-SO with different mixing ratios under different shear strains.

**Figure 23:**
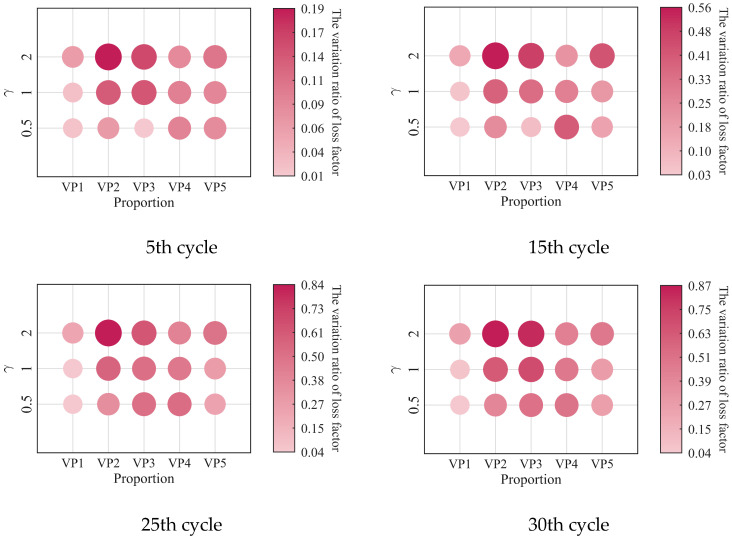
Growth ratio of the loss factor of PIB-B12-SO with different mixing ratios under different shear strains.

The variation in the low-cycle fatigue ratio with shear strain amplitude is shown in Figure 21, Figure 22 and Figure 23. At the same number of loading cycles, the low-cycle fatigue variation ratio of PIB-B12-SO increases with increasing strain amplitude. It exhibits a marginal increase with loading cycles at small strain amplitudes (0.5), but increases significantly at large strain amplitudes.

### 3.2. Recovery Property of PIB-B12-SO

**Figure 24:**
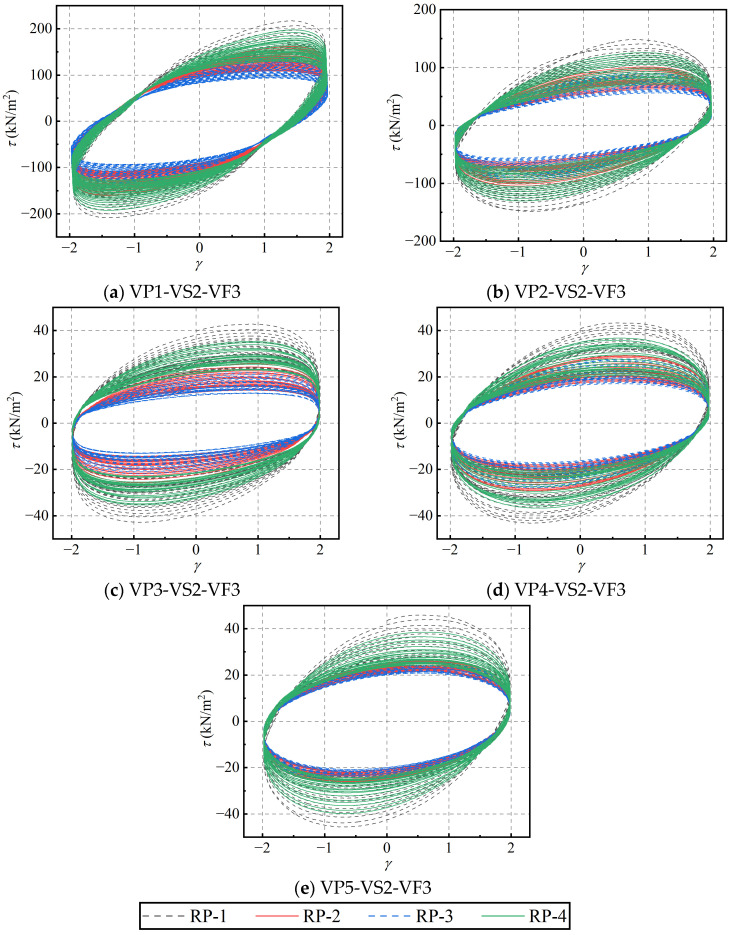
Hysteresis loops of PIB-B12-SO with different mixing ratios under different loading time intervals.

To investigate the effect of varying blend compositions on the recovery behavior of PIB-B12-SO, a multi-stage rest-recovery test was conducted at a constant loading frequency of 1.0 Hz and a strain amplitude of 2.0. Considering the time intervals between the main shock and aftershocks, the quiescent rest interval between the first and second loading stages, as well as between the second and third stages, was set to 20 min, while the interval between the third and fourth stages was extended to 24 h. The corresponding hysteresis loops of the four loading stages for different ratios are shown in Figure 24. Following a 20-min rest interval, the mechanical responses represented by the hysteresis curves decrease significantly across all specimens. However, after a 24-h rest interval, the hysteresis curves closely approach their initial states, demonstrating that the material possesses an excellent recovery capacity over long rest periods.

**Figure 25:**
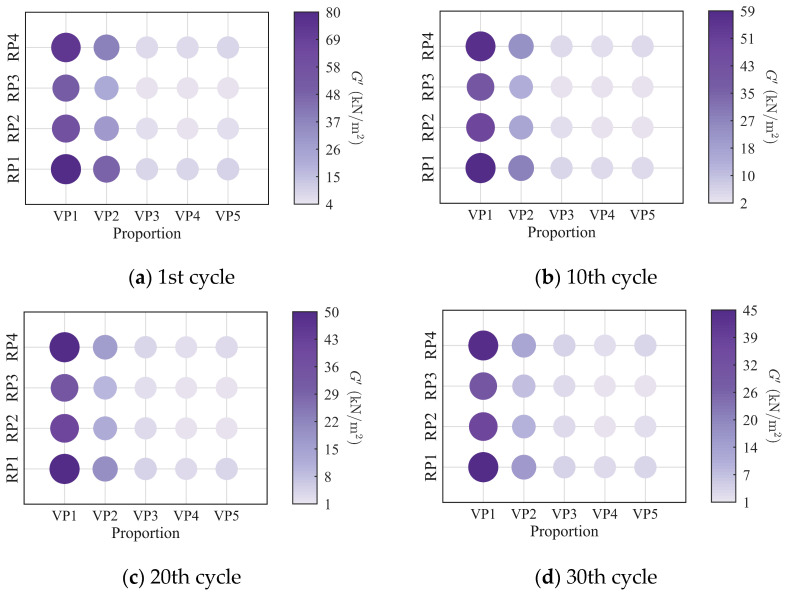
Storage modulus of PIB-B12-SO with different mixing ratios under different loading time intervals.

**Figure 26:**
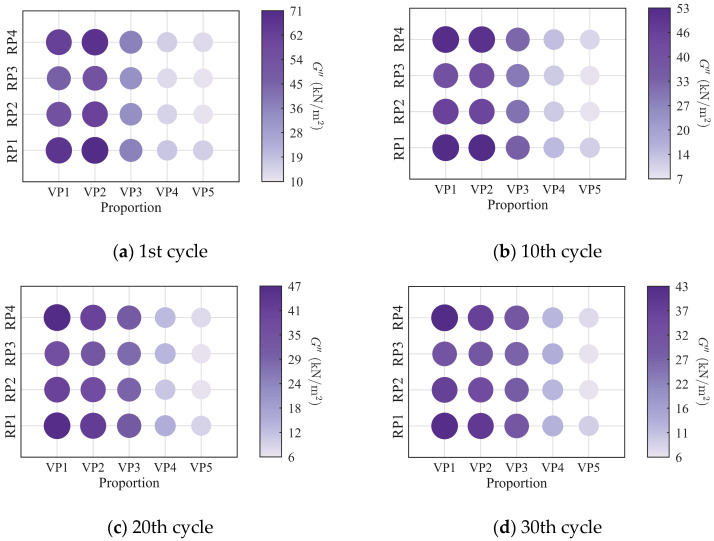
Loss modulus of PIB-B12-SO with different mixing ratios under different loading time intervals.

**Figure 27:**
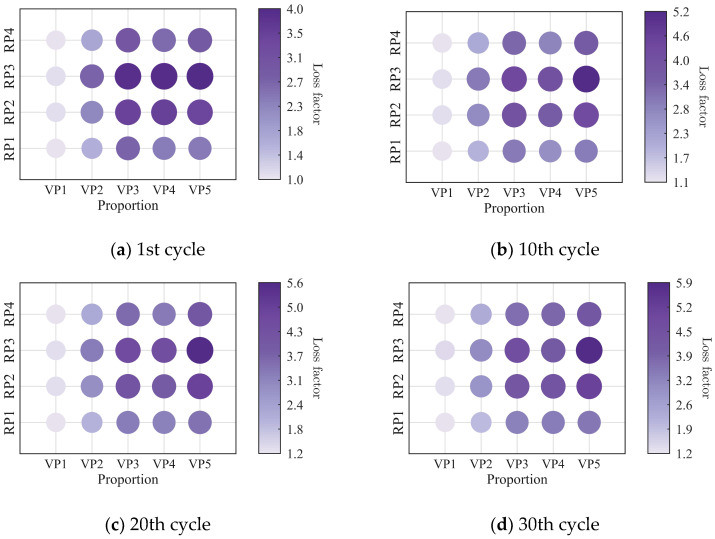
Loss factor of PIB-B12-SO with different mixing ratios under different loading time intervals.

**Figure 28:**
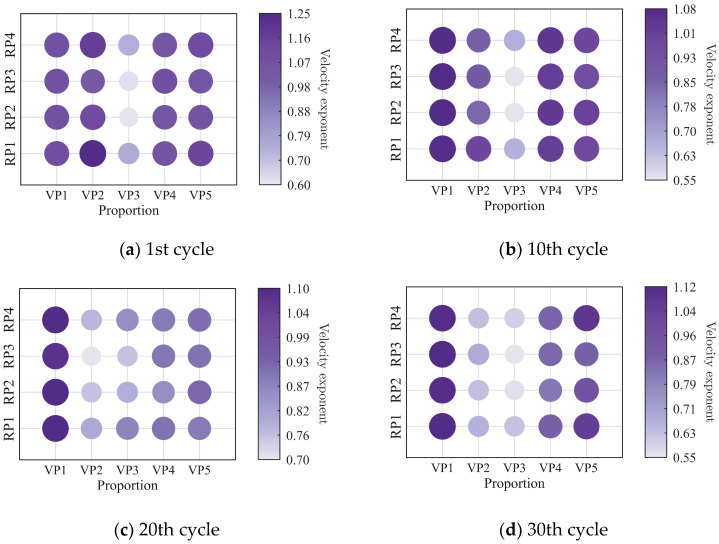
Velocity exponent of PIB-B12-SO with different mixing ratios under different loading time intervals.

The performance parameters of PIB-B12-SO with different mixing ratios under intermittent rest-recovery conditions are shown in Figure 25, Figure 26, Figure 27 and Figure 28. It can be observed that following the short quiescent rest intervals, the storage modulus, loss modulus, and velocity exponent all decrease, while the loss factor increases. In contrast, following the prolonged 24 h rest interval, the storage modulus, loss modulus, and velocity exponent increase, whereas the loss factor decreases.

The recovery coefficients of the parameters are shown in Table 3. The recovery coefficients of the storage modulus and loss modulus for different proportions range between 0 and 1, indicating that the material is capable of partial recovery within a short rest period. Notably, the coefficients obtained after the long-term rest interval (24 h) differ significantly from those after the short-term intervals (20 min). The incorporation of SO leads to an increase in the recovery coefficient of the storage modulus and the loss factor, while reducing that of the loss modulus.

Further analysis reveals that after the first and second short-term rest intervals, the loss modulus and velocity exponent recover to more than 60% of their initial values, while the storage modulus recovers to more than 50%. Conversely, given a sufficiently long rest interval (24 h), the loss modulus and velocity exponent recover to approximately 90% of their initial states, with the storage modulus reaching about 80%. These observations suggest that the presence of SO promotes the recovery of the loss modulus while moderately inhibiting the recovery of the storage modulus.

The recovery coefficient of the loss factor exhibits an increase following the short rest intervals but a relative decrease after the prolonged rest interval. Further analysis shows that the loss factor of PIB-B12-SO increases by more than 40% and 80% during the second and third loadings stages, respectively, and by more than 10% during the fourth loading stage. This occurs because, over short time intervals, the recovery of the loss modulus is stronger than that of the storage modulus, rendering the viscous response more dominant than the elastic response and consequently leading to an elevated loss factor. After long resting intervals, both the storage modulus and the loss modulus are nearly fully recovered, and the viscoelastic behavior approaches that observed during the initial loading, resulting in a reduction in the loss factor.

## 4. Discussion

### 4.1. The Evolution Model

As discussed in the preceding sections, the rheological parameters of the blends (i.e., the storage modulus, loss modulus, and velocity exponent) exhibit a significant evolution under cyclic loading. This evolution typically manifests as a rapid variation during the initial cycles, followed by a gradual stabilization as the cycling progresses. However, relying solely on discrete experimental data makes it challenging to systematically compare the low-cycle fatigue behaviors across different mixing ratios, thereby restricting their application in performance evaluation and material selection for engineering practices.

Therefore, it is imperative to parametrically characterize these experimental results. Specifically, a unified mathematical model is required to transform the cyclic response evolution into distinct phenomenological parameters with clear physical interpretations. This approach achieves three main objectives: (1) quantitatively characterizing the cyclic evolution laws; (2) facilitating a comparative analysis of the dynamic fatigue characteristics across materials with different mixing ratios; and (3) providing a reliable basis for the parametric optimization of the materials’ energy dissipation and recovery capacities. Based on the morphological features of the experimental curves, it can be observed that the variation in the material response with respect to the number of cycles exhibits a distinct asymptotic saturation characteristic; that is, a rapid initial change is followed by a gradual convergence towards a steady-state plateau. Consequently, an evolution model featuring an asymptotic limit is adopted in this study to describe the variation laws of the material parameters as a function of the number of cycles.

Under low-cycle loading, the PIB-B12-SO viscoelastic material evolves progressively, exhibiting an asymptotic saturation characteristic—a rapid initial change followed by gradual convergence toward a steady-state level. Therefore, saturation-type evolution models, such as the traditional single-exponential model, are appropriate for describing this kinetic behavior [35,36,37].

To further capture the monotonic evolution and gradual stabilization of the variation ratio of the *G*′, *G*″, and *μ* during low-cycle fatigue, a saturated stretched-exponential model is adopted in this study [38,39,40]. Let *Y*(*N*, *x*) denote the low-cycle fatigue variation ratios for a specific material parameter (*G*′, *G*″, *μ*) at a given number of cycles, *N*, and mixing ratio, *x*. Its evolution with respect to the number of cycles can be expressed as:

(15) $$Y(N,x)=Y_{m}(x)-(Y_{m}(x)-Y_{2}(x))\exp\left[-{\left(\frac{N-2}{N_{c}(x)}\right)}^{b(x)}\right]$$

where $Y_{2}(x)$ denotes the parameter level at the initial stage of cycling (around the second cycle); $Y_{m}(x)$ represents the asymptotic value during the stable cyclic stage; $N_{c}(x)$ is the characteristic number of cycles, which reflects the rate scale of the parameter’s evolution from the initial state to the steady state; and *b*(*x*) is the shape index, utilized to modulate the morphology of the evolution curve. A larger $N_{c}$ indicates a slower evolution rate of the material parameters toward the steady state, whereas *b* reflects the degree of nonlinearity in the evolution process. Specifically, when *b* approx 1, the curve approaches an exponential form; when *b* < 1, the initial variation is more rapid; and when *b* > 1, the initial variation is relatively gradual. It is worth noting that, since the number of experimental cycles is limited to 30, the parameter $Y_{m}$ partially reflects a parametric extrapolation of the long-term cyclic trend. Therefore, to ensure the stability and physical validity of the fitting results, reasonable constraints must be imposed on the parameter ranges during the model fitting process.

The fitting procedure was conducted using the constrained least squares method. The basic parameter bounds were set as $N_{c}$ > 0 and *b* > 0. Furthermore, weak constraints were imposed on $Y_{m}$ based on the physical trends (e.g., $Y_{m}\;\leq \;Y_{2}$ for degradation indicators, and $Y_{m}\;\geq {\;Y}_{2}$ for hardening indicators) to significantly mitigate the occurrence of unstable solutions resulting from the extrapolation of the short 30-cycle sequence. To comprehensively evaluate the fitting performance and explanatory capability of the proposed model, various statistical metrics were employed in this study, including RMSE, MAE, MAPE (%), NRMSE, *R*^2^, Adjusted *R*^2^, and Weighted RMSE, as detailed in Table 4.

**Figure 29:**
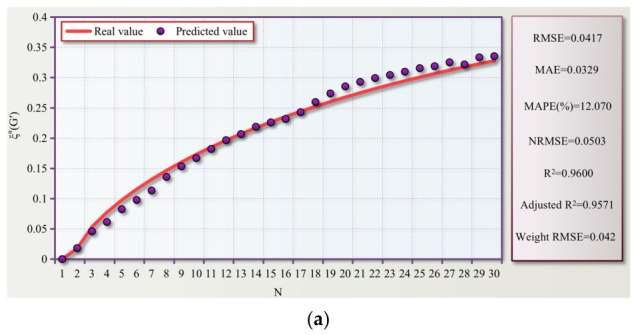

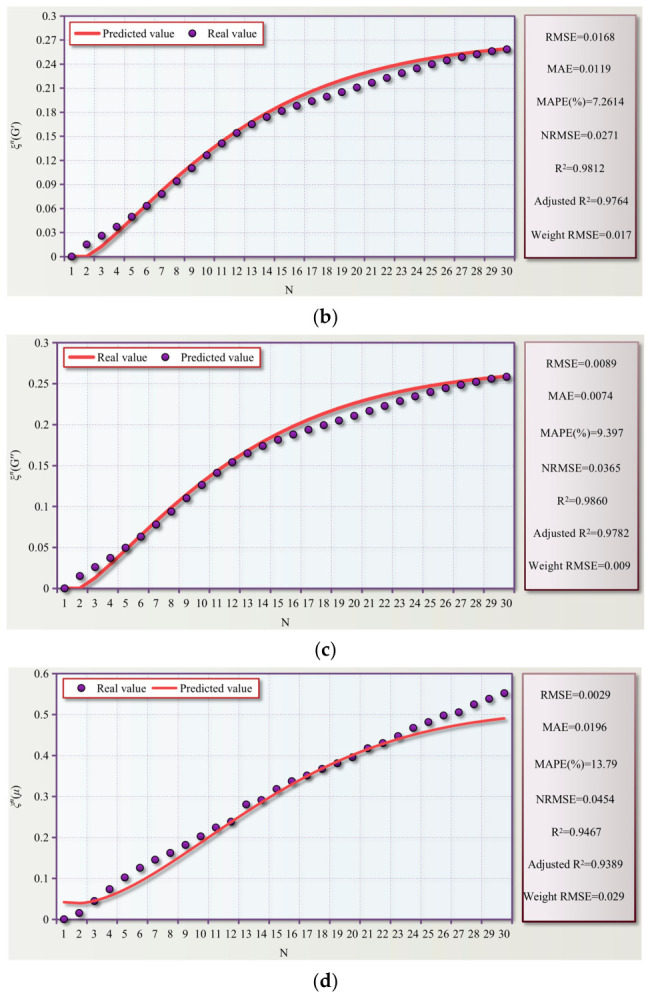
Visualized fitted curves and evaluation results for (**a**) $\xi^{n}(G^{\prime })$—VP1, (**b**) $\xi^{n}(G^{\prime })$—VP2, (**c**) $\xi^{n}(G^{\prime \prime })$—VP5, and (**d**) $\xi^{n}(\mu )$—VP2.

The prediction results of $\xi^{n}({\;G}^{\prime })\;$—VP1, $\xi^{n}({\;G}^{\prime })$—VP2, $\xi^{n}({\;G}^{\prime \prime })$—VP5, and $\xi^{n}(\mu )$ were randomly selected for visualization. The fitted curves and their corresponding evaluation metrics are illustrated in Figure 29a–d.

The visualized fitted curves and their corresponding evaluation metrics are illustrated in Figure 29. The predicted curves exhibit excellent overall agreement with the experimental data, accurately capturing both the monotonic evolution trends and the asymptotic characteristics of the parameters during the low-cycle loading process. The *R*^2^ values for all fitted groups range from 0.9467 to 0.9860, and the Adjusted *R*^2^ values range from 0.9389 to 0.9764. Analysis of the residual distribution reveals that the fitting errors are primarily concentrated in the initial transient stage where rapid macroscopic rheological variations occur, whereas the deviations remain minimal during the subsequent steady-state plateau phase. This robust statistical performance validates that the proposed model effectively reflects the kinetic characteristics of the material’s dynamic fatigue degradation. Overall, the model not only achieves high statistical fitting accuracy but also demonstrates robust stability and physical consistency in characterizing the evolutionary paths and asymptotic trends.

Furthermore, the characteristic number of cycles $N_{c}$ for materials with different mixing ratios was derived through model fitting, as presented in Table 5. This parameter reflects the time scale of performance evolution during the cyclic loading process: a larger $N_{c}$ indicates a more gradual variation in material properties during cycling, whereas a smaller Nc implies a faster convergence toward a steady state. The fitted b values are listed in Table 6. Concurrently, by processing the experimental data from the recovery phase, the recovery coefficients of the various materials at different recovery times were determined, as detailed in Table 3. This coefficient reflects the material’s capacity for performance recovery following fatigue loading, serving as a crucial indicator for evaluating its long-term service performance.

Based on the aforementioned findings, a lower variation ratio of the loss modulus during low-cycle fatigue loading enables the material to maintain a stable viscous dissipation capacity. Conversely, a higher variation ratio of the storage modulus can effectively mitigate the material’s elastic restoration characteristics, while a larger variation ratio of the loss factor characterizes an enhanced proportion of the loss component within the material’s complex response.

Therefore, the fundamental principle for optimizing the material’s low-cycle fatigue performance is to suppress the degradation of its viscous dissipation while promoting the decay of its elastic characteristics. Guided by this principle, a comprehensive index, *S*(*x*), fatigue-resistant loss performance, is formulated as expressed in Equation (16):

(16) $$S_{\max}(x)=\xi^{n}(G^{\prime }(N,x))\times \xi^{n}(\mu (N,x))/\xi^{n}(G^{\prime \prime }(N,x))$$

where $\xi^{n}(G^{\prime }(N,\;x))$ denotes the variation ratio of the loss modulus at a target number of cycles, *N*, and $\xi^{n}(\mu (N,\;x))$ represents the corresponding variation ratio of the loss factor. The index *S*(*x*) reflects the material’s capability to sustain energy dissipation during fatigue loading. A larger value indicates superior low-cycle fatigue performance, characterized by less degradation of the loss modulus.

By applying the same calculation procedure to all material mixing ratios, the corresponding energy dissipation scores were determined for each composition. According to the experimental data in Table S1, the VP3 blend yielded the highest score, with S(VP3) being the maximum among all tested ratios. These findings suggest that the VP3 composition (i.e., the 2:3 mixing ratio) provides the most robust and sustainable energy dissipation performance under low-cycle fatigue conditions and is therefore the most suitable option for VDW applications.

### 4.2. Limitation and Scope

The proposed PIB-B12-SO blend provides a highly effective and economically viable material solution for viscous damping walls (VDWs). Compared to other advanced smart materials, magnetorheological elastomers (MREs) exhibit excellent magnetic-field tunability [41,42]; however, their application in large-tonnage civil engineering dampers often requires bulky electromagnetic coils and a continuous external power supply [2]. Under extreme seismic events where power grids may fail, this reliance poses severe challenges to control robustness and introduces a high risk of systemic failure. Alternatively, incorporating rigid nanofillers (such as GO or CNTs) can effectively enhance damping through interfacial friction [43,44]. Nevertheless, while improving energy dissipation capacity, this approach typically causes a simultaneous and significant increase in the storage modulus (dynamic stiffness) [44]. For VDW applications, such excessive stiffness can introduce substantial secondary seismic forces into the primary structure. In contrast, the proposed blend demonstrates exceptional energy-absorption stability and rheological recovery capabilities under low-cycle fatigue and quiescent interval loading, making it particularly suitable for extreme seismic scenarios characterized by frequent mainshock–aftershock sequences or long-duration ground motions.

From an economic perspective, compared to existing VDWs that predominantly utilize expensive, high-performance pure silicone oil systems, the polyisobutylene and silicone oil blending strategy employed herein reduces overall material costs without compromising critical dynamic performance.

It must be acknowledged that this study possesses certain limitations regarding material characterization and theoretical modeling. First, in terms of material characterization, due to current equipment constraints, the assessment of blend homogeneity relies primarily on classical macroscopic rheological criteria (i.e., the formation of a macroscopically uniform milky-white emulsion). There is a lack of microstructural imaging to reveal the micro-evolutionary mechanisms of the morphology under varying shear histories. Second, the theoretical evolution model proposed herein currently focuses on a phenomenological description. While it effectively captures the macroscopic mechanical behavior within the tested parameter ranges, its broader generalization and predictive capabilities require further validation and refinement using entirely independent external datasets, which is currently restricted by the size of the experimental dataset.

Furthermore, the current development of viscous materials for VDWs is still largely dominated by formulation screening, lacking systematic theoretical guidance. In the future, advanced algorithms such as machine learning hold great promise for assisting in the construction of “composition–microstructure–macro-property” mapping models. This will ultimately enable the on-demand customization of viscoelastic materials tailored to specific control requirements, thereby accelerating the development cycle of novel energy dissipation media for VDWs.

## 5. Conclusions

In this study, a viscoelastic polymer blend (PIB-B12-SO) was formulated to enhance the energy dissipation stability and recovery performance of materials utilized in viscous damping walls (VDWs) under multi-cycle loading. Based on dynamic sandwich-type shear tests conducted on blends with varying mass fractions, the core findings are summarized as follows:

1. The incorporation of silicone oil (SO) significantly enhances the intrinsic energy dissipation capacity of the PIB-B12 matrix, substantially increasing the loss factor (by up to 65.6%) while maintaining a relatively low storage modulus.
2. The addition of SO effectively improves the energy-absorption stability of the material. It mitigates the degradation of the loss modulus during cyclic loading (by 16.75%), ensuring a stable and robust energy dissipation capacity over prolonged fatigue cycles.
3. The SO component remarkably accelerates quiescent recovery by restoring the loss modulus (by 13.11%) and suppressing storage modulus recovery, enabling VDWs to rapidly recover during mainshock-aftershock sequences.
4. The proposed stretched exponential evolution model effectively captures the nonlinear degradation dynamics of the macroscopic parameters under low-cycle shear loading.

## Figures and Tables

**Table 1:** The typical characteristics of PIB-B12.

Parameter	Value	Parameter	Value
Commercial grade	Oppanol-B12	Density at 23 °C	0.92 g/cm^3^
viscosity average molecular weight (*M_v_*)	55,000	Staudinger index	34.5–39.0 cm^3^/g

**Table 2:** Summary of testing conditions for PIB-B12-SO blends.

Test Case	Number	Variation Range	Purpose
Variable proportion test (PIB: SO)	VP1	1:0	Effect of proportions on storage and dissipation of materials
	VP2	5:1	
	VP3	2:3	
	VP4	1:2	
	VP5	1:4	
Variable strain test	VS1	0.5	Effect of loading strain on the low-cycle fatigue properties of materials
	VS2	1.0	
	VS3	2.0	
Variable frequency test	VF1	0.2 Hz	Effect of loading frequency on the low-cycle fatigue properties of materials.
	VF2	0.6 Hz	
	VF3	1.0 Hz	
	VF4	1.4 Hz	
	VF5	2.0 Hz	
Recovery property test	RP1	0	Effect of static recovery time on the recovery properties of materials
	RP2	20 min	
	RP3	40 min	
	RP4	24 h	

**Table 3:** Ratio of variation coefficients of PIB-B12-SO with different mixing ratios under different loading time intervals.

Case	Static Coefficient of *G*′	Static Coefficient of *G*″	Static Coefficient of Loss Factor
VP1-VS3-VF3-RP2	0.6133	0.5201	0.3007
VP1-VS3-VF3-RP3	0.8722	0.8206	0.3891
VP1-VS3-VF3-RP4	0.1948	0.1732	0.0084
VP2-VS3-VF3-RP2	0.5907	0.2980	1.5570
VP2-VS3-VF3-RP3	0.7679	0.5674	2.6469
VP2-VS3-VF3-RP4	0.2453	0.0885	0.3893
VP3-VS3-VF3-RP2	0.6471	0.5294	1.3106
VP3-VS3-VF3-RP3	0.9490	0.7676	1.8785
VP3-VS3-VF3-RP4	0.2157	0.1059	0.4369
VP4-VS3-VF3-RP2	0.7639	0.6223	1.1700
VP4-VS3-VF3-RP3	0.8403	0.7431	1.5211
VP4-VS3-VF3-RP4	0.2344	0.1444	0.2099
VP5-VS3-VF3-RP2	0.6676	0.5649	0.9383
VP5-VS3-VF3-RP3	0.9200	0.7842	1.3989
VP5-VS3-VF3-RP4	0.2736	0.1505	0.4265

**Table 4:** Evaluation metrics for model fitting.

Abbreviation	Formula	Description
RMSE	$\mathrm{RMSE}=\sqrt{\frac{1}{n}\sum_{i=1}^{n}{\left(y_{i}-{\hat{y}}_{i}\right)}^{2}}$	Root mean squared error
MSE	$\mathrm{MAE}=\frac{1}{n}\sum_{i=1}^{n}\left|y_{i}-{\hat{y}}_{i}\right|$	Mean squared error
MAPE(%)	$\mathrm{MAPE}(\%)=\frac{100}{n}\sum_{i=1}^{n}\left|\frac{y_{i}-{\hat{y}}_{i}}{y_{i}}\right|$	Mean Absolute Percentage Error (%)
NRMSE	${\mathrm{NRMSE}}_{\mathrm{range}}=\frac{\mathrm{RMSE}}{y_{\max}-y_{\min}}$	Normalized Root Mean Squared Error
*R* ^2^	$R^{2}=1-\frac{\sum_{i=1}^{n}{\left(y_{i}-{\hat{y}}_{i}\right)}^{2}}{\sum_{i=1}^{n}{\left(y_{i}-\bar{y}\right)}^{2}}$	Coefficient of Determination
Adjusted *R*^2^	$R_{\mathrm{adj}}^{2}=1-(1-R^{2})\frac{n-1}{n-p-1}$	Adjusted Coefficient of Determination
Weighted RMSE	$\mathrm{wRMSE}=\sqrt{\frac{\sum_{i=1}^{n}\omega_{i}{\left(y_{i}-{\hat{y}}_{i}\right)}^{2}}{\sum_{i=1}^{n}\omega_{i}}}$	Weighted Root Mean Squared Error

**Table 5:** Reference values of *N_c_*.

Proportion	*N*_c_ ($\xi^{n}(G^{\prime })$)	*N*_c_ ($\xi^{n}(G^{\prime \prime })$)	*N*_c_ ($\xi^{n}(\mu )$)
VP1	26.8286	12.1381	16.9755
VP2	13.6669	11.4468	14.5271
VP3	31.8583	10.9379	14.7307
VP4	50.6446	11.0116	15.5767
VP5	167.097	11.2837	18.1819

**Table 6:** Reference values of *b*.

Parameter	*b* ($\xi^{n}(G^{\prime })$)	*b* ($\xi^{n}(G^{\prime \prime })$)	*b* ($\xi^{n}(\mu )$)
Value	0.7774	1.25584	1.66308

## Data Availability

The data presented in this study are not publicly available but may be available from the corresponding authors upon reasonable request.

## Supplementary Materials

The following supporting information can be downloaded at: https://www.mdpi.com/article/10.3390/polym18091022/s1, Table S1: The parameters of the low-cycle fatigue test; Table S2: The parameters of the recovery test.
