# Supplementary material for: Experimental Study of Low-Cycle Fatigue and Recovery of Polymer Blends for Viscous Damping Walls
Source: Polymers (Basel). 2026 Apr 23;18(9):1022. doi: 10.3390/polym18091022 (PMC13164962; doi:10.3390/polym18091022)
Supplement: Supplementary file 1 [file polymers-18-01022-s001.zip › polymers-4251641-supplementary.pdf]

Table S1 The parameters of the low-cycle fatigue test

$\xi^n(G')$						$\xi^n(G'')$					$\xi^n(\mu)$				
N	VP1	VP2	VP3	VP4	VP5	VP1	VP2	VP3	VP4	VP5	VP1	VP2	VP3	VP4	VP5
1	0.000	0.000	0.000	0.000	0.000	0.000	0.000	0.000	0.000	0.000	0.000	0.000	0.000	0.000	0.000
2	0.018	0.053	0.070	0.035	0.046	0.016	0.042	0.033	0.020	0.015	0.011	0.015	0.046	0.024	0.021
3	0.046	0.106	0.146	0.072	0.080	0.037	0.067	0.065	0.036	0.026	0.021	0.044	0.072	0.041	0.037
4	0.061	0.154	0.221	0.110	0.113	0.055	0.091	0.096	0.053	0.037	0.026	0.073	0.097	0.058	0.053
5	0.082	0.198	0.276	0.133	0.142	0.077	0.111	0.125	0.068	0.050	0.032	0.102	0.122	0.076	0.068
6	0.098	0.240	0.320	0.172	0.169	0.094	0.129	0.154	0.083	0.063	0.037	0.126	0.145	0.095	0.082
7	0.113	0.277	0.365	0.200	0.193	0.104	0.144	0.181	0.097	0.078	0.044	0.145	0.168	0.115	0.096
8	0.136	0.311	0.419	0.223	0.216	0.116	0.157	0.204	0.112	0.094	0.050	0.162	0.190	0.135	0.110
9	0.153	0.343	0.455	0.257	0.238	0.131	0.169	0.224	0.127	0.110	0.053	0.182	0.212	0.156	0.125
10	0.167	0.372	0.494	0.287	0.259	0.146	0.180	0.240	0.144	0.126	0.055	0.202	0.232	0.177	0.141
11	0.182	0.401	0.528	0.309	0.277	0.160	0.190	0.253	0.160	0.141	0.057	0.224	0.253	0.198	0.155
12	0.197	0.428	0.553	0.342	0.292	0.175	0.199	0.264	0.176	0.154	0.058	0.238	0.275	0.220	0.165
13	0.207	0.454	0.590	0.355	0.303	0.187	0.208	0.273	0.191	0.165	0.061	0.255	0.301	0.242	0.172
14	0.219	0.476	0.617	0.370	0.310	0.198	0.216	0.281	0.205	0.174	0.064	0.265	0.331	0.262	0.175
15	0.226	0.497	0.633	0.387	0.317	0.209	0.224	0.289	0.218	0.181	0.069	0.289	0.365	0.280	0.177
16	0.231	0.517	0.674	0.411	0.323	0.220	0.232	0.298	0.231	0.188	0.071	0.306	0.403	0.296	0.180
17	0.243	0.535	0.675	0.437	0.330	0.230	0.239	0.306	0.242	0.194	0.073	0.319	0.442	0.309	0.185
18	0.259	0.550	0.703	0.461	0.340	0.240	0.246	0.315	0.254	0.199	0.075	0.334	0.479	0.318	0.194
19	0.274	0.565	0.721	0.467	0.352	0.251	0.252	0.323	0.264	0.205	0.076	0.346	0.511	0.326	0.207
20	0.285	0.578	0.745	0.473	0.364	0.258	0.258	0.331	0.272	0.211	0.077	0.360	0.538	0.332	0.220
21	0.293	0.590	0.756	0.483	0.375	0.265	0.263	0.338	0.280	0.217	0.077	0.380	0.559	0.339	0.232
22	0.299	0.601	0.773	0.495	0.385	0.269	0.268	0.344	0.285	0.223	0.081	0.391	0.578	0.347	0.243
23	0.304	0.612	0.784	0.533	0.392	0.275	0.273	0.350	0.290	0.229	0.082	0.407	0.594	0.355	0.251
24	0.310	0.622	0.794	0.539	0.399	0.278	0.278	0.355	0.294	0.234	0.083	0.425	0.608	0.365	0.258
25	0.315	0.631	0.803	0.564	0.406	0.283	0.284	0.359	0.298	0.240	0.084	0.438	0.621	0.375	0.264
26	0.319	0.640	0.813	0.596	0.413	0.287	0.289	0.363	0.303	0.244	0.084	0.452	0.634	0.385	0.271
27	0.325	0.649	0.825	0.612	0.420	0.293	0.295	0.367	0.307	0.248	0.089	0.469	0.646	0.395	0.277
28	0.322	0.658	0.834	0.620	0.427	0.294	0.302	0.370	0.312	0.252	0.089	0.486	0.657	0.406	0.284
29	0.333	0.667	0.844	0.630	0.433	0.300	0.308	0.374	0.317	0.256	0.091	0.508	0.668	0.416	0.290
30	0.335	0.674	0.847	0.622	0.440	0.302	0.316	0.377	0.323	0.258	0.093	0.520	0.678	0.428	0.297

Table S2 The parameters of the recovery test

$\psi^n(G')$						$\psi^n(G'')$					$\psi^n(\mu)$				
N	VP1	VP2	VP3	VP4	VP5	VP1	VP2	VP3	VP4	VP5	VP1	VP2	VP3	VP4	VP5
RP2	0.613	0.591	0.647	0.764	0.668	0.520	0.298	0.529	0.622	0.565	0.301	1.557	1.311	1.170	0.938
RP3	0.872	0.768	0.949	0.840	0.920	0.821	0.567	0.768	0.743	0.784	0.389	2.647	1.879	1.521	1.399
RP4	0.195	0.245	0.216	0.234	0.274	0.173	0.089	0.106	0.144	0.151	0.008	0.389	0.437	0.210	0.427
